# The relationship between nutritional status and prealbumin levels in children with loss of appetite and iron deficiency: a prospective cross-sectional study

**DOI:** 10.3389/fnut.2025.1647870

**Published:** 2025-09-16

**Authors:** Ömer Okuyan, Sinem Durmus, Hafize Uzun

**Affiliations:** ^1^Department of Pediatrics, Istanbul Atlas University Hospital, Faculty of Medicine, Istanbul Atlas University, Istanbul, Türkiye; ^2^Department of Medical Biochemistry, Faculty of Medicine, İzmir Katip Çelebi University, İzmir, Türkiye; ^3^Department of Medical Biochemistry, Faculty of Medicine, Istanbul Atlas University, Istanbul, Türkiye

**Keywords:** children, loss of appetite, controlling nutritional status, prealbumin, prognostic nutritional index, nutritional risk index

## Abstract

**Background:**

Prealbumin, a hepatic protein with a short half-life, has emerged as a sensitive biomarker for assessing nutritional status. This study aimed to evaluate the relationship between nutritional status and prealbumin levels in children experiencing loss of appetite due to iron deficiency and to compare the diagnostic value of prealbumin with established nutritional indices.

**Methods:**

A total of 260 children aged 2–15 years were enrolled and categorized into four groups: control (*n* = 65), appetite loss only (*n* = 65), iron deficiency only (*n* = 65), and both conditions (*n* = 65). Nutritional status was assessed using laboratory biomarkers and indices such as the Controlling Nutritional Status (CONUT) score, Prognostic Nutritional Index (PNI), and Nutritional Risk Index (NRI). Prealbumin levels were measured using the enzyme-linked immunosorbent assay (ELISA).

**Results:**

Prealbumin levels were significantly lower in all patient groups than in controls (*p* < 0.001). Prealbumin showed strong positive correlations with total cholesterol (*r* = 0.528), albumin (*r* = 0.489), and PNI (*r* = 0.489), and a strong negative correlation with the CONUT score (*r* = −0.546) (all *p* < 0.001). ROC analysis demonstrated that prealbumin had high diagnostic accuracy in distinguishing appetite loss (AUC = 0.911) and iron deficiency (AUC = 0.892). Logistic regression confirmed that prealbumin was significantly associated with both conditions (*p* < 0.001), whereas other indices (CONUT, PNI, and NRI) were not.

**Conclusion:**

While appetite loss is a clinically reported symptom, reduced prealbumin levels reflect the underlying nutritional impact of this condition. Prealbumin is a sensitive and reliable biomarker for assessing nutritional deterioration associated with both appetite loss and iron deficiency in children and outperforms commonly used nutritional indices. Its use may improve early recognition and management of nutritional risk in pediatric populations. Further research is needed to explore the diagnostic and prognostic value of nutritional indices (CONUT, PNI, and NRI) in children.

## Introduction

Loss of appetite is a non-specific nutritional problem that can accompany a variety of different problems. It is frequently observed in children with iron deficiency and may contribute to deterioration in nutritional status. Appetite loss not only reduces caloric intake but also decreases the consumption of essential nutrients, which may further exacerbate deficiencies and compromise overall health (1).

Iron deficiency is one of the most common nutritional disorders in children worldwide and a leading cause of anemia. It is particularly prevalent in developing countries but remains a public health concern globally. Iron plays a critical role in various physiological processes, including oxygen transport, cellular respiration, and immune function. In children, iron deficiency can significantly impair cognitive development, growth, and overall health (2).

Prealbumin migrates to the anode more rapidly than albumin. It is also known as thyroxine-binding prealbumin (TBPA) or transthyretin because it binds thyroxine and triiodothyronine. Structurally, prealbumin is a homotetrameric protein composed of four identical subunits, allowing it to bind and transport thyroid hormones and retinol-binding protein. With a circulating half-life of approximately 12 h, the rate of synthesis of prealbumin is proportional to the synthesis capacity of the liver and the dietary intake of the necessary substrates, making it one of the best markers of the body’s nutritional status. Prealbumin, a hepatic protein with a short half-life, has emerged as a sensitive biomarker for assessing nutritional status. It reflects recent dietary protein intake and is more responsive to acute changes in nutritional status compared to other proteins, such as albumin. However, the role of prealbumin in pediatric populations, particularly in children with appetite loss and iron deficiency, remains underexplored (3, 4).

Nutritional indices such as the Controlling Nutritional Status (CONUT) score, Prognostic Nutritional Index (PNI), and Nutritional Risk Index (NRI) are commonly used in clinical settings to evaluate the nutritional status of patients (5–8). While these indices incorporate biochemical and anthropometric parameters, their applicability in pediatric populations and in the context of appetite loss and iron deficiency requires further investigation.

Prealbumin is a hepatic protein with a short half-life (2–3 days), making it a sensitive marker of recent nutritional status. Compared to albumin, it reflects acute changes more rapidly and is less affected by hydration or chronic conditions. Its use may support early detection of nutritional compromise, particularly in children with appetite loss and/or iron deficiency, and guide timely intervention in clinical practice. To the best of our knowledge, no previous studies have evaluated prealbumin, CONUT, PNI, or NRI specifically in pediatric populations with appetite loss and iron deficiency, making our findings a novel contribution to the literature. This study aimed to examine the relationship between nutritional status and prealbumin levels in children with loss of appetite. By evaluating the association between prealbumin and other biochemical markers, as well as established nutritional indices, this study sought to identify the most reliable indicators of nutritional compromise in this vulnerable population. In addition, it aimed to determine the diagnostic performance of prealbumin in distinguishing between appetite loss and iron deficiency.

## Materials and methods

### Study design and population

The protocol for sample collection was approved by the Istanbul Atlas University, Atlas University Hospital, Clinical Research Ethics Committee (date: 17 February 2025; E-22686390-050.99-6187). The study was performed in accordance with the 1975 Helsinki Declaration, updated in 2013. Written informed consent was obtained from the parents or legal guardians of all participating children.

Children aged 2–15 years who met all inclusion criteria were consecutively recruited from the pediatric outpatient clinics of Atlas University Hospital. This recruitment method was chosen to reduce selection bias and to ensure that all eligible patients presenting during routine clinical care were systematically considered for enrollment.

In this study, patients were categorized into four distinct groups according to the presence or absence of iron deficiency and appetite loss:

Control group: no iron deficiency and no appetite loss.
Iron deficiency (ID) group: iron deficiency without appetite loss.
Appetite loss group: appetite loss without iron deficiency.
Combined group: both iron deficiency and appetite loss are present.

### Inclusion criteria

(i) Children aged 2–15 years. (ii) Diagnosed with iron deficiency based on laboratory parameters (e.g., low serum ferritin, low serum iron, and elevated total iron-binding capacity). (iii) Children presenting with loss of appetite as reported by caregivers or based on clinical evaluation. (iv) Availability of complete laboratory data, including prealbumin, albumin, total protein, cholesterol levels, and other nutritional biomarkers. (v) Informed consent obtained from parents or legal guardians.

### Exclusion criteria

(i) Presence of chronic diseases such as chronic kidney disease, liver disease, malignancy, or autoimmune disorders. (ii) Children with acute infections or inflammatory conditions at the time of sampling. (iii) Use of iron supplements or other nutritional support within the past 3 months. (iv) Diagnosed with malabsorption syndromes (e.g., celiac disease and cystic fibrosis). (v) History of genetic or hematological disorders affecting iron metabolism (e.g., thalassemia, sickle cell anemia). (vi) Incomplete medical records or missing laboratory data required for analysis. (vii) Children with known chronic liver disease and acute infections at the time of admission were excluded to reduce the potential confounding effects of inflammation and hepatic dysfunction on prealbumin levels. (viii) To minimize confounding factors, children with known chronic systemic diseases, recent infections, or hospitalizations within the last 3 months were excluded from the study.

In this study, we evaluated the appetite status and feeding behavior of children with a scale in terms of ease of applicability and strength of objectivity. When we examined the scales developed for this purpose, we preferred the “Children’s Eating Behavior Questionnaire” (CEBQ), which is the Turkish adaptation of the “Children’s Eating Behavior Questionnaire,” because it is comprehensive, objective, and has proven validity and reliability in terms of evaluating children’s feeding behaviors (9, 10).

### Laboratory parameters

From all patients, a 2-mL blood sample was collected into a K3 EDTA tube (Vacuette; Greiner Bio-One, Kremsmünster, Austria) for a complete blood count (CBC) parameter, whereas a 3-mL blood sample was drawn into standard tubes for biochemical parameters, ferritin, iron, and total iron-binding capacity (TIBC) assays. The result of CBC was recorded with an automatic hematology analyzer (Sysmex XN-1000, Norderstedt, Germany). Routine biochemical parameters in blood were measured with an automated analyzer (COBAS 8000, ROCHE-2007, Tokyo, Japan). Serum prealbumin levels were measured by the enzyme-linked immunosorbent assay (ELISA; Assaypro, Saint Charles, MI, United States). Ferritin concentration was analyzed by immunoassay using an automated chemiluminescence (Immulite 2000; Diagnostic Products Corporation, LA, United States). Serum iron and TIBC were measured by an autoanalyzer (Olympus AU 1600; Mishima, Japan). The transferrin saturation was calculated from the formula: (serum iron/TIBC) × 100.

### Nutritional indices

The Prognostic Nutritional Index (PNI), Controlling Nutritional Status (CONUT) score, and Nutritional Risk Index (NRI) were calculated to assess nutritional status.

- PNI was calculated as: 10 × serum albumin (g/dL) + 0.005 × total lymphocyte count (/mm^3^). Lower values indicate poorer nutritional status (11).
- CONUT was determined based on serum albumin, total lymphocyte count, and total cholesterol (TC) levels, with higher scores (0–12) reflecting greater nutritional impairment (12).
- NRI was calculated as: [1.519 × serum albumin (g/dL)] + [41.7 × actual weight (kg)/ideal body weight (IBW)]. IBW was estimated using the Lorentz formula. Lower NRI scores indicate higher nutritional risk. Body mass index (BMI) was calculated and will be classified according to the World Health Organization (WHO). A lower NRI indicates a higher risk of malnutrition (13–15).

### Statistical analysis

The sample size was determined by power analysis (power = 80%, effect size = 0.5, *α* = 0.05), resulting in 65 participants per group. Normality was assessed using the Shapiro–Wilk test. Group comparisons were performed using one-way ANOVA with Bonferroni *post-hoc* tests for normally distributed data, followed by the Student’s *t*-test or Mann–Whitney *U*-test as appropriate. ROC analysis was used for diagnostic accuracy, and multivariate logistic regression assessed associations between variables. A *p*-value of <0.05 was considered statistically significant. All data were analyzed using JASP 0.19.1 (University of Amsterdam, Netherlands) software.

## Results

**Figure 1:**
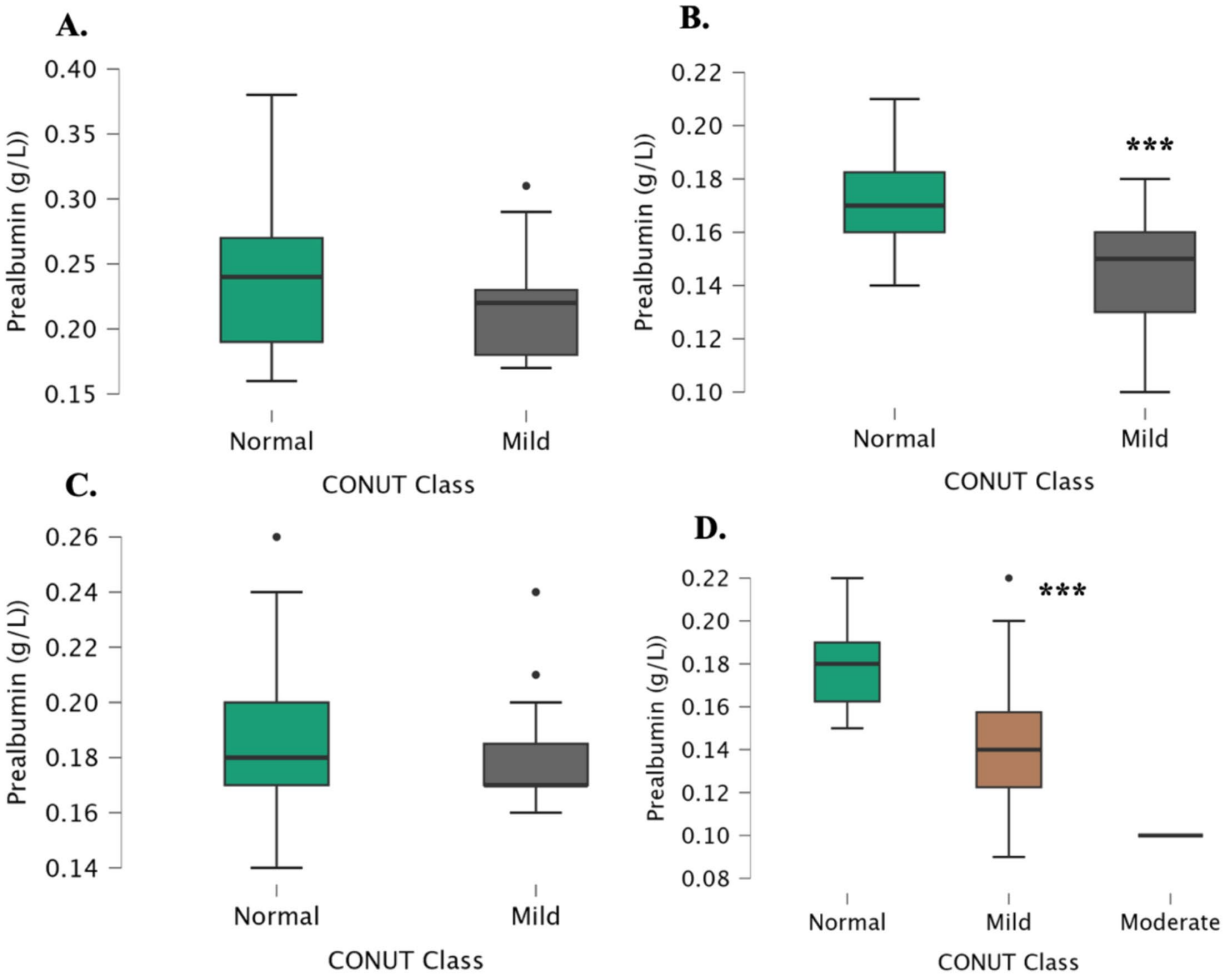
Prealbumin levels across subgroups based on CONUT score in different study groups. **(A)** Control, **(B)** with appetite loss, **(C)** with early iron deficiency, **(D)** with iron deficiency and appetite loss.

Baseline characteristics, including age and sex, were compared across the four study groups to assess homogeneity. Statistical analysis revealed no significant differences among the groups in these variables (*p* > 0.05), indicating that the groups were comparable at baseline. This ensured that observed differences in nutritional or biochemical parameters were not confounded by major demographic imbalances. Demographic and laboratory characteristics of the study groups are presented in Table 1. When examining the alternative subgroups of CONUT scores, prealbumin levels did not differ significantly between the normal and mild CONUT score groups in both the control group and the early iron deficiency group (Figures 1A,C). However, in children with appetite loss, prealbumin levels were significantly lower in those with a mild CONUT score compared to those with a normal CONUT score (*p* < 0.001) (Figure 1B). Additionally, in children with both iron deficiency and appetite loss, prealbumin levels were significantly lower in the mild CONUT score group (*p* < 0.001) (Figure 1D).

**Table 1:** Demographic and laboratory characteristics of the study groups.

Characteristic	Control (*n* = 65)	With appetite loss (*n* = 65)	With early iron deficiency (*n* = 65)	With iron deficiency and appetite loss (*n* = 65)
Age (years)	6.79 ± 3.68	6.62 ± 3.29	7.05 ± 3.86	6.40 ± 3.04
Gender^#1^ Female Male	43 (66.15%) 22 (33.85%)	34 (52.31%) 31 (47.69%)	30 (46.15%) 35 (53.85%)	33 (50.77%) 32 (49.23%)
BMI (kg/m^2^)	18.57 ± 3.34	17.02 ± 3.91	17.53 ± 3.29	17.41 ± 5.01
Hb (g/dL)	12.27 ± 0.96	10.80 ± 0.85 a***	10.46 ± 0.78 a***	10.40 ± 0.78 a***, b*
Hct (%)	39.97 ± 1.37	35.75 ± 2.28 a***	36.40 ± 2.98 a***	34.89 ± 2.42 a***, c**
Total cholesterol (mg/dL)	163.71 ± 30.69	144.97 ± 43.73 a*	155.85 ± 37.26	141.70 ± 45.75 a**
LDL (mg/dL)	87.40 ± 27.72	89.03 ± 17.87	90.56 ± 20.98	85.66 ± 22.20
HDL (mg/dL)	51.57 ± 11.79	53.09 ± 13.22	52.96 ± 9.94	55.30 ± 13.56
VLDL (mg/dL)	12.17 ± 2.46	13.66 ± 7.52	16.75 ± 11.64 a**	14.05 ± 8.55
Total protein (g/dL)	7.40 ± 0.46	7.07 ± 0.67 a**	7.41 ± 0.48 b**	7.09 ± 0.64 a*, c*
Albumin (g/dL)	4.81 ± 0.32	4.59 ± 0.30 a*	4.80 ± 0.54	4.59 ± 0.35 a*, c*
Prealbumin (mg/dL)	0.24 ± 0.05	0.16 ± 0.02 a***	0.19 ± 0.03 a***, b**	0.16 ± 0.03 a***, c**
Total iron-binding capacity (TIBC) (ng/mL)	291.51 ± 45.65	320.54 ± 89.14	278.03 ± 60.13 b**	273.25 ± 56.86 b***
Iron (μg/dL)	76.59 ± 21.44	60.71 ± 25.02	72.20 ± 71.51	67.55 ± 30.83
Ferritin (ng/mL)	43.46 ± 27.84	34.15 ± 24.25	33.04 ± 21.29	39.20 ± 36.20
CONUT score^#2^ Normal Mild Moderate	52 (80.00%) 13 (20.00%) 0 (N.A.)	40 (61.54%) 25 (38.46%) 0 (N.A.)	53 (81.54%) 12 (18.46%) 0 (−)	34 (52.31%) 30 (46.15%) 1 (1.54%)
PNI score	48.09 ± 3.21	45.96 ± 3.00 a*	48.05 ± 5.35 b*	45.94 ± 3.51 a*, c*
NRI score	47.03 ± 7.84	43.95 ± 9.04 a**	45.52 ± 8.76	44.39 ± 7.65 a*

a: vs. control, b: vs. with appetite loss c: vs. with early iron deficiency.

#1: means *p* = 0.121 and #2: means *p* = 0.002 as chi-square tests.

Hb, hemoglobin; Hct, hematocrit; TIBC, total iron-binding capacity; CONUT, Controlling Nutritional Status; PNI, Prognostic Nutritional Index; NRI, Nutritional Risk Index.

Correlation analysis showed positive and significant relationships between prealbumin levels and TC (*r* = 0.528; *p* < 0.001), albumin (*r* = 0.489; *p* < 0.001), and PNI (*r* = 0.489; *p* < 0.001), while a negative relationship was observed between prealbumin and CONUT score (*r* = −0.546; *p* < 0.001). Strong positive relationships were found between TC and albumin (*r* = 0.566; *p* < 0.001) and PNI (*r* = 0.566; *p* < 0.001), while a negative relationship was found with the CONUT score (*r* = −0.744, *p* < 0.001). A negative relationship between albumin and CONUT score (*r* = −0.461; *p* < 0.001) and a strong positive relationship between PNI and CONUT score (*r* = 1.000; *p* < 0.001) were observed. No significant relationship was found between CONUT score and NRI (*r* = −0.034; *p* = 0.790). A positive but non-significant relationship was found between the PNI and the NRI (*r* = 0.220; *p* = 0.078).

**Figure 2:**
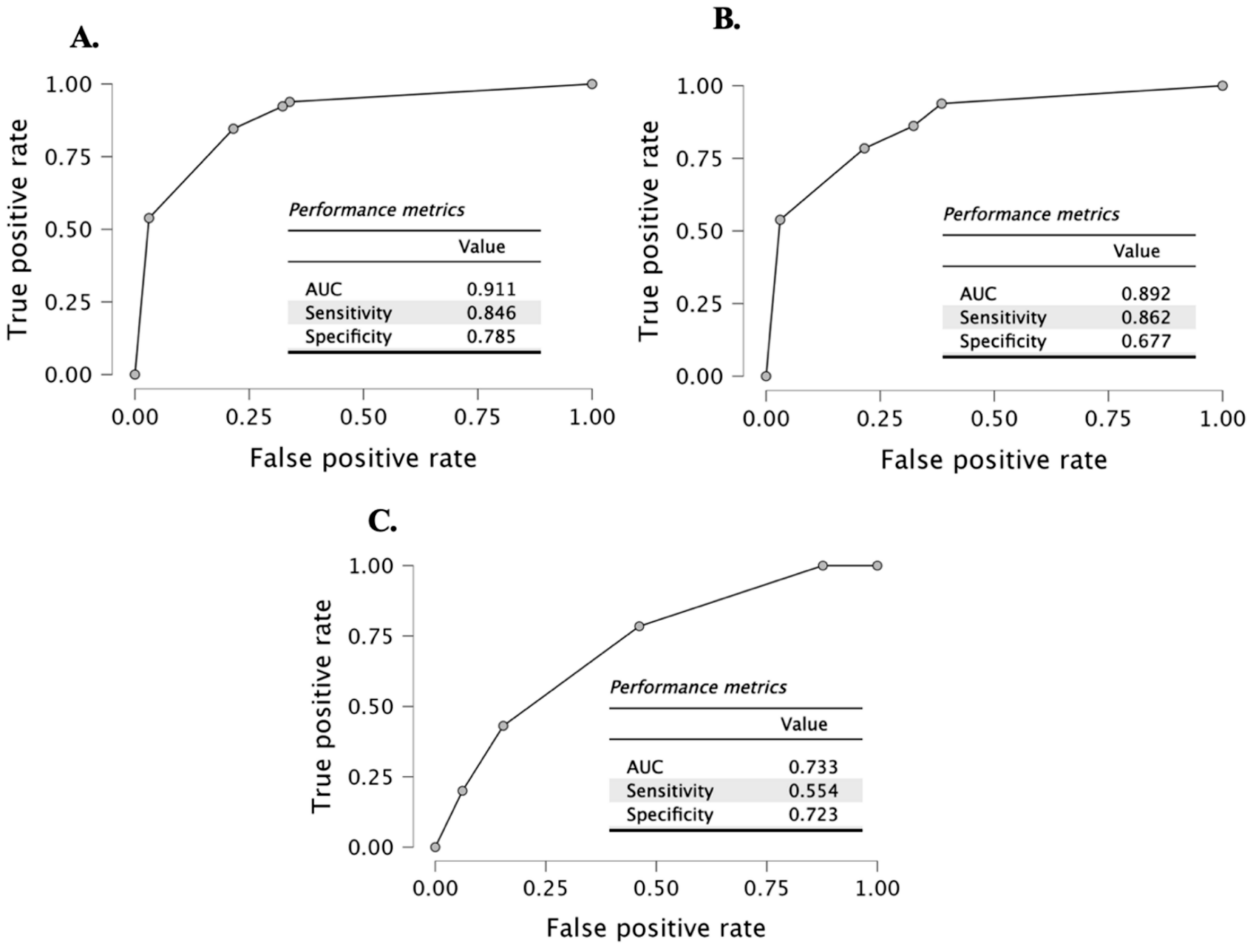
ROC curve **(A)** control vs. with appetite loss; **(B)** control vs. with appetite loss and with iron deficiency; and **(C)** with appetite loss vs. with early iron deficiency groups.

ROC analysis showed that prealbumin levels had a high capacity to differentiate between the iron deficiency and children with appetite loss conditions from the control group. The ability to differentiate between iron deficiency and children with appetite loss was found to have an area under the curve (AUC) of 0.892 (95% CI = 0.835–0.949), sensitivity of 0.862, and specificity of 0.677 (Figure 2B). For differentiating only children with appetite loss, the AUC = 0.911 (95% CI = 0.859–0.963), sensitivity = 0.846, and specificity = 0.785 were found (Figure 2A). Finally, the ability to differentiate between children with appetite loss and early iron deficiency was evaluated, and the AUC = 0.733 (95% CI = 0.647–0.819), sensitivity = 0.554, and specificity = 0.723 were found (Figure 2C).

In this study, logistic regression analysis was performed to evaluate the relationship between appetite loss, iron deficiency, and various biomarkers across three different groups. The first analysis examined the differences between the control group and children with appetite loss. The results revealed a strong negative relationship between prealbumin levels and appetite loss. In Model M_0_, each unit decrease in prealbumin levels was associated with an increased likelihood of appetite loss (*p* = 1.381 × 10^−7^, odds ratio = 4.395 × 10^−15^). However, other nutritional indicators such as CONUT, PNI, and NRI did not show a significant relationship with appetite loss (*p* > 0.05) (Table 2).

**Table 2:** Logistic regression analysis results between the control and children with appetite loss.

Model		Odds ratio	*z*	*p*	95% confidence interval	
					Lower bound	Upper bound
M_0_	(Intercept)	139503.795	5.427	5.733 × 10^−8^	7.568	16.124
	Prealbumin (g/L)	4.311 × 10^−28^	−5.268	1.381 × 10^−7^	−86.456	−39.567
M_1_	(Intercept)	1.657 × 10^+9^	3.423	6.193 × 10^−4^	9.073	33.383
	Prealbumin (g/L)	1.051 × 10^−29^	−4.992	5.976 × 10^−7^	−92.923	−40.527
	CONUT	0.670	−1.153	0.249	−1.080	0.280
	PNI	0.853	−1.351	0.177	−0.391	0.072
	NRI	0.985	−0.449	0.653	−0.084	0.052

In the second analysis, the differences between children with appetite loss and iron deficiency were examined. Prealbumin levels were again found to be significantly associated with iron deficiency, with each unit increase in prealbumin levels significantly increasing the risk of iron deficiency (*p* = 1.825 × 10^−5^, odds ratio (OR) = 4.008 × 10^17^). This finding suggests that prealbumin levels may serve as an important biomarker in diagnosing iron deficiency. Other nutritional parameters, such as CONUT, PNI, and NRI, did not show a significant relationship with iron deficiency (*p* > 0.05) (Table 3).

**Table 3:** Logistic regression analysis results between the control and early iron deficiency.

Model		Odds ratio	*z*	*p*	95% confidence interval	
					Lower bound	Upper bound
M_0_	(Intercept)	873.596	5.160	2.470 × 10^−7^	4.200	9.345
	Prealbumin (g/L)	4.395 × 10^−15^	−5.078	3.808 × 10^−7^	−45.817	−20.300
M₁	(Intercept)	385.987	2.110	0.035	0.424	11.487
	Prealbumin (g/L)	2.017 × 10^−15^	−5.058	4.242 × 10^−7^	−46.949	−20.725
	CONUT	0.861	−0.574	0.566	−0.661	0.362
	PNI	1.031	0.597	0.551	−0.070	0.130
	NRI	0.993	−0.275	0.784	−0.057	0.043

Finally, the relationship between appetite loss and iron deficiency was examined. This analysis also found a strong positive relationship between prealbumin levels and iron deficiency (*p* = 2.080 × 10^−4^). However, no significant relationship was observed between other nutritional indices, such as CONUT, PNI, and NRI, with iron deficiency (*p* > 0.05). These results indicate that prealbumin levels are the strongest biomarker in determining the presence of iron deficiency (Table 4).

**Table 4:** Logistic regression analysis results between children with appetite loss and iron deficiency.

Model		Odds ratio	*z*	*p*	95% confidence interval	
					Lower bound	Upper bound
M_0_	(Intercept)	8.921 × 10^−4^	−4.276	1.904 × 10^−5^	−10.241	−3.803
	Prealbumin (g/L)	4.008 × 10^+17^	4.285	1.825 × 10^−5^	21.994	59.070
M₁	(Intercept)	8.524 × 10^−6^	−2.884	0.004	−19.606	−3.739
	Prealbumin (g/L)	8.020 × 10^+15^	3.709	2.080 × 10^−4^	17.269	55.972
	CONUT	0.977	−0.101	0.920	−0.474	0.428
	PNI	1.115	1.386	0.166	−0.045	0.263
	NRI	1.005	0.234	0.815	−0.040	0.050

## Discussion

This study demonstrated that prealbumin levels were significantly reduced in children with appetite loss, iron deficiency, or both. Prealbumin showed strong positive correlations with albumin, total cholesterol, hemoglobin (Hb), and hematocrit (Hct), and a strong negative correlation with the CONUT score. ROC analysis confirmed that prealbumin had high diagnostic accuracy in identifying nutritional risk, outperforming conventional indices such as CONUT, PNI, and NRI, which showed limited sensitivity in the pediatric population. Prealbumin emerges as a sensitive and reliable biomarker for early detection of nutritional compromise in children, particularly in the context of appetite loss and iron deficiency. Its integration into routine clinical assessment may enhance early identification of at-risk patients. However, it should be interpreted alongside clinical evaluation and additional laboratory parameters to ensure a comprehensive nutritional assessment. Prealbumin testing is indeed accessible and cost-effective within the public health system of our region. It is routinely performed in hospital laboratories with a relatively low cost and rapid turnaround time compared to more comprehensive nutritional assessments. This makes prealbumin a practical and valuable tool for early nutritional screening in pediatric patients, especially in settings where resources may be limited.

In our cohort, prealbumin levels were significantly lower in children with appetite loss, iron deficiency, or both conditions compared to healthy controls. The lowest prealbumin levels were observed in children with both appetite loss and iron deficiency, indicating a cumulative negative impact on nutritional status. This is consistent with existing literature demonstrating that iron deficiency adversely affects appetite and overall nutritional intake (16, 17). Moreover, iron deficiency is known to influence appetite-regulating hormones such as ghrelin and leptin, contributing to reduced food intake and further nutritional compromise (18, 19). Our findings align with these data, supporting the physiological interplay between iron metabolism and appetite regulation.

Clinical evidence indicates that iron supplementation can lead to notable improvements in appetite among children with iron deficiency anemia. This relationship was clearly demonstrated in the study by Granero et al. (20), which reported increased food intake and improved subjective appetite ratings in children who received iron treatment compared to those given a placebo. One of the important clinical observations in this study is the association between iron deficiency and poor appetite, which supports previous literature identifying reduced appetite as a hallmark symptom of iron deficiency. We found significant positive correlations between prealbumin and other key nutritional markers such as albumin, total cholesterol, and PNI, alongside a significant negative correlation with the CONUT score. These results reinforce the role of prealbumin as a sensitive marker for protein-energy malnutrition and immune status, as described in previous studies (21–24). ROC curve analysis demonstrated the strong discriminative ability of prealbumin for identifying children at nutritional risk, with AUC values exceeding 0.89, supporting its diagnostic accuracy reported in neonatal and pediatric intensive care studies (23, 24). These findings support previous studies that highlight the diagnostic utility of prealbumin in pediatric populations for both nutritional assessment and disease severity stratification (21–25). Megied et al. (25) suggested that prealbumin may not reliably reflect malnutrition or predict outcomes in critically ill children, highlighting the need for larger, longitudinal studies to clarify its role in severe pediatric illness. In children with cancer, hypoalbuminemia and low prealbumin levels may indicate inflammation-related malnutrition and predict a higher risk of infectious complications during treatment, highlighting the importance of early nutritional interventions (26). While C-reactive protein (CRP)-to-prealbumin and CRP-to-albumin ratios may enhance the predictive value of individual biomarkers for mortality and severe disease-related malnutrition, their limited standalone accuracy underscores the need for further studies and combined clinical interpretation (27). Combining prealbumin with inflammatory markers such as CRP or interleukin-6 (IL-6) could improve its diagnostic accuracy by helping to differentiate malnutrition from inflammation-induced hypo-prealbuminemia. This approach has been proposed in recent literature to enhance the clinical utility of prealbumin in both pediatric and adult populations (21, 22). Although prealbumin is associated with recent dietary intake, its reliability as a follow-up biomarker in the pediatric intensive care unit is limited due to its strong inverse correlation with inflammation markers like CRP rather than nutritional intake alone (28). Our findings, which demonstrate a strong association between low prealbumin levels and both appetite loss and iron deficiency in children, are consistent with previous studies showing that prealbumin reflects nutritional status and can serve as a sensitive marker for growth and disease severity in pediatric populations. Although we excluded patients with acute infections and liver dysfunction, it is important to acknowledge that prealbumin remains a negative acute-phase reactant, and subclinical inflammation or unrecognized hepatic factors may still influence its levels to some extent.

Nutritional status reflects general health, including immune competence, protein turnover, and physical condition (29). Tools such as CONUT, PNI, and NRI are widely used to screen for malnutrition risk. The CONUT score is calculated using serum albumin, TC, and lymphocyte count values typically available in routine labs and reflects both protein and calorie reserves (30–32). The PNI, another immuno-nutritional index, uses albumin and lymphocyte levels (30), while the NRI incorporates serum albumin and weight relative to ideal body weight (31).

In our study, children with appetite loss and mild CONUT scores had significantly lower prealbumin levels than those with normal CONUT scores. Similarly, in children with both appetite loss and iron deficiency, prealbumin levels were lower among those with mild CONUT scores. Correlation analyses showed positive associations between prealbumin and albumin, TC, and PNI, and a negative association with CONUT. These findings align with prior studies demonstrating prealbumin’s sensitivity to nutritional and immune status (33, 34). Logistic regression confirmed that low prealbumin levels were strongly associated with appetite loss and iron deficiency, while CONUT, PNI, and NRI were not.

Additionally, although CONUT includes TC, a marker of caloric reserve, it did not significantly correlate with iron deficiency in our cohort. This suggests that prealbumin is a more sensitive marker than these indices in early nutritional risk detection among pediatric patients, consistent with observations in elderly and cancer populations (34). Unlike prealbumin, nutritional indices such as CONUT, PNI, and NRI did not show significant associations with appetite loss or iron deficiency in this pediatric population. This may be because these indices were originally developed for adult populations and may not sensitively reflect early or mild nutritional changes in children. In contrast, prealbumin has a shorter half-life and responds rapidly to nutritional fluctuations, making it a more sensitive marker in pediatric settings. This discrepancy suggests the need for pediatric-specific validation of conventional nutritional indices. These tools, although validated primarily in adults with ischemic stroke, cancer, and metabolic diseases (5, 35–37), may lack sensitivity in children due to differences in physiological and metabolic profiles. This observation concurs with prior literature highlighting the need for pediatric-specific nutritional assessment tools, as adult-derived indices may underestimate malnutrition risk in children (34). The CONUT score includes TC as an indicator of calorie reserves; however, its adult-based reference ranges may limit its applicability in children (32, 33). Our data, consistent with findings in elderly cancer populations (34), suggest that prealbumin provides a more sensitive indication of early protein-energy malnutrition than CONUT and other indices in pediatric settings. Zhang et al. (35) similarly highlighted that the PNI may underestimate malnutrition compared to CONUT and NRI in elderly patients, raising concerns about the direct translation of these indices to pediatric care.

This study has several limitations. First, potential confounding factors such as socioeconomic status, detailed dietary intake, and recent mild infections could not be comprehensively evaluated. These variables may influence both appetite and nutritional biomarkers, including prealbumin. Second, although we excluded patients with overt infections or liver disease based on clinical and laboratory evaluations, subclinical inflammation or hepatic dysfunction might still have had a minor effect on prealbumin levels, which is known to be a negative acute-phase reactant. Third, although the use of objective indices such as CONUT, PNI, and NRI offers standardized assessments, their validity and cutoff points in pediatric populations require further large-scale validation.

In conclusion, our study supports prealbumin as a sensitive, cost-effective, and practical biomarker for early nutritional assessment in children with appetite loss and/or iron deficiency. While conventional indices such as CONUT, PNI, and NRI remain useful, their limited performance in pediatric patients highlights the need for tailored nutritional screening approaches. Incorporating prealbumin measurement into routine clinical practice may facilitate timely nutritional interventions, potentially improving pediatric health outcomes. Further larger-scale prospective pediatric studies are needed to validate these findings and to explore the prognostic value of prealbumin and other nutritional biomarkers, including potential confounding variables, in this vulnerable population.

## Data Availability

The raw data supporting the conclusions of this article will be made available by the authors, without undue reservation.

## References

[1] Kral TVE Moore RH Chittams J O’Malley L Jones E Quinn RJ. Caloric compensation and appetite control in children of different weight status and predisposition to obesity. Appetite. (2020) 151:104701. doi: 10.1016/j.appet.2020.104701, PMID: 32289325 PMC7305978

[2] Kucuk N Orbak Z Karakelloglu C Akcay F. The effect of therapy on plasma ghrelin and leptin levels, and appetite in children with iron deficiency anemia. J Pediatr Endocrinol Metab. (2019) 32:275–80. doi: 10.1515/jpem-2018-0352, PMID: 30796846

[3] Keller U. Nutritional laboratory markers in malnutrition. J Clin Med. (2019) 8:775. doi: 10.3390/jcm8060775, PMID: 31159248 PMC6616535

[4] Liz MA Coelho T Bellotti V Fernandez-Arias MI Mallaina P Obici L. A narrative review of the role of transthyretin in health and disease. Neurol Ther. (2020) 9:395–402. doi: 10.1007/s40120-020-00217-0, PMID: 33001386 PMC7606379

[5] Misirlioglu NF Uzun N Ozen GD Çalik M Altinbilek E Sutasir N. The relationship between neutrophil-lymphocyte ratios with nutritional status, risk of nutritional indices, prognostic nutritional indices and morbidity in patients with ischemic stroke. Nutrients. (2024) 16:1225. doi: 10.3390/nu16081225, PMID: 38674915 PMC11054104

[6] Nelms CL Shaw V Greenbaum LA Anderson C Desloovere A Haffner D. Assessment of nutritional status in children with kidney diseases-clinical practice recommendations from the Pediatric Renal Nutrition Taskforce. Pediatr Nephrol. (2021) 36:995–1010. doi: 10.1007/s00467-020-04852-5, PMID: 33319327 PMC7910229

[7] Hongya D Linfan D Chunyuan H Jun J Bin L Jian Z. Prognostic nutritional index enhances the discriminatory ability of procalcitonin for predicting pediatric sepsis. Glob Pediatr Health. (2024) 11:2333794X241245277. doi: 10.1177/2333794X241245277, PMID: 38606322 PMC11008342

[8] Çetin İD Çetin O. A preliminary study on the association between prognostic nutritional index and neutrophil-to-lymphocyte ratio with nutritional status and inflammation in febrile children’s susceptibility to seizures. Rev Assoc Med Bras. (2024) 70:e20240166. doi: 10.1590/1806-9282.20240166, PMID: 39045938 PMC11262348

[9] Wardle JGC Sanderson S Rapoport L. Development of the children’s eating behaviour questionnaire. J Child Psychol Psychiatry. (2001) 42:963–70. doi: 10.1111/1469-7610.00792, PMID: 11693591

[10] Malczyk Ż Kuczka O Pasztak-Opiłka A Zachurzok A. Validation of the children’s eating behaviour questionnaire in Poland. Nutrients. (2022) 14:4782. doi: 10.3390/nu14224782, PMID: 36432467 PMC9693564

[11] Nishikawa H Yoh K Enomoto H Ishii N Iwata Y Takata R. The relationship between controlling nutritional (CONUT) score and clinical markers among adults with hepatitis C virus related liver cirrhosis. Nutrients. (2018) 10:1185. doi: 10.3390/nu10091185, PMID: 30158477 PMC6164819

[12] Iseki Y Shibutani M Maeda K Nagahara H Ohtani H Sugano K. Impact of the preoperative controlling nutritional status (CONUT) score on the survival after curative surgery for colorectal cancer. PLoS One. (2015) 10:e0132488. doi: 10.1371/journal.pone.0132488, PMID: 26147805 PMC4492767

[13] Bo Y Wang K Liu Y You J Cui H Zhu Y. The geriatric nutritional risk index predicts survival in elderly esophageal squamous cell carcinoma patients with radiotherapy. PLoS One. (2016) 11:e0155903. doi: 10.1371/journal.pone.0155903, PMID: 27196126 PMC4873221

[14] Al-Najjar Y Clark AL. Predicting outcome in patients with left ventricular systolic chronic heart failure using a nutritional risk index. Am J Cardiol. (2012) 109:1315–20. doi: 10.1016/j.amjcard.2011.12.026, PMID: 22335857

[15] Onodera T Goseki N Kosaki G. Prognostic nutritional index in gastrointestinal surgery of malnourished cancer patients. Nihon Geka Gakkai Zasshi. (1984) 85:1001–5. 6438478

[16] Lozoff B Beard J Connor J Barbara F Georgieff M Schallert T. Long-lasting neural and behavioral effects of iron deficiency in infancy. Nutr Rev. (2006) 64:S34–43. doi: 10.1301/nr.2006.may.S34-S43, PMID: 16770951 PMC1540447

[17] Wessells KR Brown KH. Estimating the global prevalence of zinc deficiency: results based on zinc availability in national food supplies and the prevalence of stunting. PLoS One. (2012) 7:e50568. doi: 10.1371/journal.pone.0050568, PMID: 23209782 PMC3510072

[18] Sachdev HS Gera T Nestel P. Effect of iron supplementation on mental and motor development in children: systematic review of randomised controlled trials. Public Health Nutr. (2006) 8:117–32. doi: 10.1079/phn2004677 15877905

[19] Pasricha SR Black J Muthayya S Shet A Bhat V Nagaraj S. Determinants of anemia among young children in rural India. Pediatrics. (2013) 131:e462–70. doi: 10.1542/peds.2009-3108 20547647

[20] Granero R Pardo-Garrido A Carpio-Toro IL Ramírez-Coronel AA Martínez-Suárez PC Reivan-Ortiz GG. The role of iron and zinc in the treatment of ADHD among children and adolescents: a systematic review of randomized clinical trials. Nutrients. (2021) 13:4059. doi: 10.3390/nu13114059, PMID: 34836314 PMC8618748

[21] Dellière S Cynober L. Is transthyretin a good marker of nutritional status? Clin Nutr. (2017) 36:364–70. doi: 10.1016/j.clnu.2016.06.004, PMID: 27381508

[22] Ranasinghe RN Biswas M Vincent RP. Prealbumin: the clinical utility and analytical methodologies. Ann Clin Biochem. (2022) 59:7–14. doi: 10.1177/0004563220931885, PMID: 32429677

[23] Kim DH Lee NM Kim SY Yi DY Yun SW Chae SA. Effectiveness of prealbumin as an indicator of growth in neonates. Medicine. (2021) 100:e27603. doi: 10.1097/MD.0000000000027603, PMID: 34678912 PMC8542146

[24] Shenkin A. Serum prealbumin: is it a marker of nutritional status or of risk of malnutrition? Clin Chem. (2006) 52:2177–9. doi: 10.1373/clinchem.2006.077412, PMID: 17138848

[25] Megied MAAE Ayada IK Dayem OYAE El Wareth RAENA Ghonaim MS Mohamed AO. Diagnostic and prognostic utility of prealbumin as a nutritional biomarker in critically ill children: a prospective cross sectional study. Egypt Pediatr Assoc Gaz. (2023) 71:54. doi: 10.1186/s43054-023-00202-w

[26] Milaniuk A Drabko K Chojęta A. Role of albumin and prealbumin in assessing nutritional status and predicting increased risk of infectious complications during childhood cancer treatment. Acta Biochim Pol. (2024) 71:13693. doi: 10.3389/abp.2024.13693, PMID: 39619813 PMC11604468

[27] García-Moreno RM Mola Reyes L López-Plaza B Palma Milla S. C-reactive protein-to-prealbumin and C-reactive protein-to-albumin ratios as nutritional and prognostic markers in hospitalized patients-an observational study. Nutrients. (2024) 16:2610. doi: 10.3390/nu16162610, PMID: 39203747 PMC11357292

[28] Tekgüç H Özel D Sanaldi H Akbaş H Dursun O. Prealbumin and retinol binding proteins are not usable for nutrition follow-up in pediatric intensive care units. Pediatr Gastroenterol Hepatol Nutr. (2018) 21:321–8. doi: 10.5223/pghn.2018.21.4.321, PMID: 30345246 PMC6182478

[29] Więch P Sałacińska I Bączek M Bazaliński D. The nutritional status of healthy children using bioelectrical impedance and anthropometric measurement. J Pediatr. (2022) 98:161–7. doi: 10.1016/j.jped.2021.05.009, PMID: 34242586 PMC9432148

[30] Huang ZZ Song CG Huang JJ Xia W Bi XW Hua X. Prognostic significance of the controlling nutritional status (CONUT) score in surgically treated breast cancer patients. Gland Surg. (2020) 9:1370–9. doi: 10.21037/gs-20-294, PMID: 33224812 PMC7667092

[31] Barge-Caballero E García-López F Marzoa-Rivas R Barge-Caballero G Couto-Mallón D Paniagua-Martín MJ. Prognostic value of the nutritional risk index in heart transplant recipients. Rev Esp Cardiol. (2017) 70:639–45. doi: 10.1016/j.rec.2017.01.005, PMID: 28209304

[32] Gadgil MD Anderson CA Kandula NR Kanaya AM. Dietary patterns are associated with metabolic risk factors in South Asians living in the United States. J Nutr. (2015) 145:1211–7. doi: 10.3945/jn.114.207753, PMID: 25904730 PMC4442115

[33] Ignacio de Ulibarri J Gonzalez-Madrono A de Villar NG González P González B Mancha A. CONUT: a tool for controlling nutritional status. First validation in a hospital population. Nutr Hosp. (2005) 20:38–45. PMID: 15762418

[34] Li SY Wan LL Liu YF Li YW Huang X Liu RJ. Prognostic value of three clinical nutrition scoring system (NRI, PNI, and CONUT) in elderly patients with prostate cancer. Front Nutr. (2024) 11:1436063. doi: 10.3389/fnut.2024.1436063, PMID: 39410925 PMC11473420

[35] Zhang Q Qian L Liu T Ding JS Zhang X Song MM. Prevalence and prognostic value of malnutrition among elderly cancer patients using three scoring systems. Front Nutr. (2021) 8:738550. doi: 10.3389/fnut.2021.738550, PMID: 34708064 PMC8544751

[36] Matysiak K Hojdis A Szewczuk M. Controlling nutritional status (CONUT) score as prognostic indicator in stage IV gastric cancer with chronic intestinal failure. Nutrients. (2024) 16:4052. doi: 10.3390/nu16234052, PMID: 39683445 PMC11643072

[37] Yamanaka-Kohno R Shirakawa Y Inoue-Minakuchi M Yokoi A Noma K Tanabe S. Association between change in prognostic nutritional index during neoadjuvant therapy and dental occlusal support in patients with esophageal cancer under neoadjuvant therapy: a retrospective longitudinal pilot study. Nutrients. (2024) 16:4383. doi: 10.3390/nu16244383, PMID: 39771004 PMC11679835

